# Factors associated with domestic violence in pregnant women during the COVID-19 pandemic: Araraquara Cohort study

**DOI:** 10.1192/bjb.2024.43

**Published:** 2025-08

**Authors:** Leonardo Domingos Biagio, Delanjathan Devakumar, Leticia Falcão de Carvalho, Natália Pinheiro de Castro, Rossana Verónica Mendoza López, Liania Alves Luzia, Perla Pizzi Argentato, Patrícia Helen Carvalho Rondó

**Affiliations:** 1 School of Public Health, University of São Paulo, Brazil; 2 Institute for Global Health, University College London, UK; 3 São Paulo State Cancer Institute (ICESP), Brazil

**Keywords:** Domestic violence, pregnant women, mental health, COVID-19, pandemic

## Abstract

**Aims and method:**

This cross-sectional study, carried out from 2021 to 2022, investigated the factors associated with domestic violence in 400 Brazilian pregnant women during the COVID-19 pandemic. Violence was assessed with the World Health Organization's Violence Against Women questionnaire and the Abuse Assessment Screen. Demographic, socioeconomic, obstetric, lifestyle and mental health data were collected.

**Results:**

Violence at any time in their lives was reported by 52.2% of the women, and psychological violence was the most prevalent type (19.5%). Violence was associated with being single and mental health changes. Pregnant women exposed to any lifetime violence and psychological violence were, respectively, 4.67 and 5.93 times more likely to show mental health changes compared with women with no reported violence.

**Clinical implications:**

Training health professionals involved in prenatal care in the early detection of single women and women with mental health changes could be important in preventing domestic violence.

## The COVID-19 pandemic

As a consequence of the worldwide COVID-19 pandemic, the World Health Organization (WHO) recommended social distancing measures to minimise cases and avoid overburdening health services.^1^ However, for women in abusive relationships, staying at home was not safe, and may have increased the risk of domestic violence alongside reducing access to support networks.

Social distancing during the pandemic has probably increased the domestic work of many women, including caring for children and the elderly. Consequently, men perceived to have less power in the domestic environment, which may be one of the causes for violent behaviour; in addition, the aggressor spent more time with the victim at home.^2,3^

## Risk factors for domestic violence

The most common risk factors for domestic violence are economic condition, education, age, unwanted pregnancy, stressful experiences, history of depression, exposure to violence during childhood, marital status, lack of social support, and alcohol and drug consumption.^4,5^ The prevalence of domestic violence increased during the pandemic in some countries, and the magnitude of the problem may be even greater since the number of cases is widely underestimated, particularly in underprivileged countries. Moreover, the prevalence of domestic violence may continue to increase even after the pandemic, because of unemployment and financial instability, with the loss of income making victims more dependent on the abuser.^6^

Prenatal care is a window of opportunity to assess violence, since the victim regularly attends health services.^7^ However, limited access to antenatal care has become a reality during the pandemic, particularly for vulnerable women who live in rural areas or precarious settlements. Negative consequences, such as maternal and neonatal mortality and the risk of unwanted pregnancies, have increased during the pandemic.^8,9^

This study aimed to assess the factors associated with domestic violence in Brazilian pregnant women during the COVID-19 pandemic.

## Method

This cross-sectional study, carried out from February 2021 to August 2022, investigated the factors associated with domestic violence in 400 Brazilian pregnant women during the COVID-19 pandemic. The study is part of an ongoing large, prospective epidemiological study, the Araraquara Cohort study.

The pregnant women were selected by trained interviewers at the 34 health units in Araraquara City, São Paulo, Brazil, and were included in the study if they had a gestational age of ≤26 weeks. The participants answered a questionnaire previously tested in pregnant women,^10^ which consisted of demographic and socioeconomic (age, race, marital status and educational level), lifestyle (smoking and alcohol consumption), obstetric (gestational age and parity) and morbidity characteristics. The women attended the municipal maternity of Araraquara for ultrasound measurements before 20 weeks’ gestation, to confirm their gestational age. The participants were asked about violence and mental health changes, using standardised questionnaires.

The study was conducted in accordance with the guidelines of the Declaration of Helsinki for research involving human participants, and was approved by the Ethics Committee on Human Research of the Faculty of Medicine, University of São Paulo (protocol number 59787216.2.0000.5421). Written informed consent was obtained from all participants before data collection.

Before starting a conversation about violence, the interviewer was instructed to ask the participant if it were safe to speak and if she could simply answer ‘yes’ or ‘no’. If the women felt unsafe, the interviewer suggested a better time for the interview. In addition, the interviewer asked the participant if she was alone, to ensure that the perpetrator was not in the same room. At the end of the interviews, the pregnant women received information and contacts of women's protection services, including free psychological care in Araraquara city. The coping strategies were reinforced and the information was shared to help other women in the same situation.

### Questionnaires for assessing violence

#### World Health Organization Violence Against Women questionnaire

The World Health Organization Violence Against Women questionnaire (WHO-VAW)^11^ consists of 13 items that assess spousal violence (mental, physical and sexual) in the past 12 months, and has been used in several international and national studies, including a study of pregnant women in Brazil.^12,13^

#### Abuse Assessment Screen

The Abuse Assessment Screen (ASS)^14^ contains five questions designed to identify the frequency and severity of events, the locations of the injuries suffered and the profile of the perpetrator. The questions assess lifetime experiences of abuse, physical violence in the past year, physical violence during pregnancy, sexual abuse in the past 12 months and fear of a current partner or someone close to the woman. The AAS has been applied to Brazilian pregnant women to assess violence during pregnancy^.15^

### Questionnaires for assessing mental health

#### General Health Questionnaire

The General Health Questionnaire (GHQ)^16^ version used in this study included 12 questions and the GHQ score was classified as low (0–3) and high (≥4). The GHQ examines changes in psychological well-being in the adult population, including pregnant women,^17^ and has been validated in Brazil^.18^

#### Patient Health Questionnaire-9

The Patient Health Questionnaire-9 (PHQ-9)^19^ consists of nine symptoms and aims to measure the risk of major depression. The frequency of each symptom in the past 2 weeks is rated on a Likert scale from 0 to 3. The total score ranges from 0 to 27, and a score ≥10 is defined as risk of depression. The instrument has been validated for Brazilian adults,^20^ and also applied to Brazilian pregnant women.^21^

### Statistical analysis

Descriptive analysis was performed to obtain the frequency and percentage of the variables. The chi-squared test was used to evaluate the associations of any lifetime violence and psychological violence with maternal age, race, marital status, education, per capita income, head of household, alcohol consumption, smoking, use of illicit drugs, parity, gestational age and mental health. Variables showing statistically significant associations were included in two multivariable logistic regression models that considered any lifetime violence and psychological violence in the past 12 months as dependent variables. Results were expressed as odds ratios and 95% confidence intervals. Statistical analysis was performed with the SPSS for Windows version 20.0 software (SPSS, Chicago, USA), and a *P*-value <0.05 was adopted as statistically significant.

## Results

A total of 471 pregnant women were invited to participate in the study; 71 (15.1%) of them declined to participate for two main reasons: 29 (6.2%) had no interest and 42 (8.9%) had a lack of time (due to work and caring for children). Therefore, 400 women were included in the study.

It was necessary to reschedule the interview for three pregnant women, in view that they did not felt safe to speak or were not alone at home.

The demographic, socioeconomic, obstetric and lifestyle characteristics, violence exposure, and mental health of the pregnant women are shown in Table 1. Most of the women were aged 20–30 years (87%), non-White (52%), married or cohabiting (87.5%), had ≤12 years of schooling (81%) and had a per capita income of 0.5–1 Brazilian minimum wage (where 1 is equal to US$267.00) (56.3%). Approximately 41% of the women had an intimate partner as the head of the household. The majority of the women did not consume alcohol (86.7%), did not smoke (93.5%) and did not use illicit drugs (98%) during pregnancy. Almost 43% of the women were primiparous and 78.7% were between 14 and 26 weeks of gestation.

**Table 1:** Demographic, socioeconomic, obstetric and lifestyle characteristics, violence and mental health of the pregnant women

Characteristics	*n* (%)
Age (years)	
<20	30 (7.5)
20–30	348 (87)
>30	22 (5.5)
Race	
White	192 (48)
Black, mixed race, Asian or Indigenous	208 (52)
Marital status	
Married/common-law union	350 (87.5)
Single/divorced/widow	50 (12.5)
Education (years)	
≤12	324 (81)
>12	76 (19)
Per capita income^a^	
<0.5	148 (37)
0.5–1	225 (56.3)
>1	27 (6.8)
Head of household	
Intimate partner	162 (40.5)
Pregnant woman	73 (18.2)
Intimate partner and pregnant woman	88 (22)
Relatives	77 (19.2)
Alcohol consumption	
Yes	53 (13.2)
No	347 (86.7)
Smoking	
Yes	26 (6.5)
No	374 (93.5)
Use of illicit drugs	
Yes	8 (2)
No	392 (98)
Parity	
0	171 (42.8)
1–2	190 (47.5)
≥3	39 (9.8)
Gestational age (weeks)	
≤13	85 (21.6)
14–26	315 (78.7)
AAS	209 (52.2)
WHO-VAW	
Psychological	78 (19.5)
Physical	30 (7.5)
Sexual	11 (2.7)
GHQ score	
0–3	229 (57.3)
≥4	171 (42.7)
PHQ-9 score	
0–9	161 (40.3)
≥10	239 (59.7)

AAS, Assessment Abuse Screen; WHO-VAW, World Health Organization Violence Against Women; GHQ, General Health Questionnaire; PHQ-9, Patient Health Questionnaire-9.

a. Minimum Brazilian wage of 1 is equal to US$267.

Violence experienced at any time in their lives was reported by 52.2% of the pregnant women, and psychological violence in the past 12 months was the most prevalent type of domestic violence (19.5%). According to the GHQ and PHQ-9 scores, 42.7 and 59.7% of pregnant women exhibited mental health changes, respectively.

As can be seen in Table 2, experiencing any lifetime violence was associated with mental health changes (*P* < 0.001), psychological violence (*P* < 0.001), physical violence (*P* < 0.001), sexual violence (*P* = 0.009), age (*P* = 0.03), marital status (*P* = 0.001) and parity (*P* = 0.01). According to Table 3, psychological violence in the past 12 months was associated with mental health changes (*P* < 0.001), any lifetime violence (*P* < 0.001), physical violence (*P* < 0.001), sexual violence (*P* < 0.001), marital status (*P* < 0.001) and illicit drug use (*P* = 0.028).

**Table 2:** Associations of any lifetime violence with demographic, socioeconomic, obstetric and lifestyle characteristics, types of violence in the past 12 months and mental health of the pregnant women

Variable	No	Yes	*P*-value
Age (years)			
<20	20 (10.5%)	8 (3.8%)	0.030
20–30	146 (76.4%)	175 (83.7%)	
>30	25 (13.1%)	26 (12.4%)	
Marital status			
Married/common-law union	178 (93.2%)	172 (82.3%)	0.001
Single/divorced/widow	13 (6.8%)	37 (17.7%)	
Parity			
0	90 (47.1%)	81 (38.8%)	0.010
1–2	91 (47.9%)	99 (47.4%)	
≥3	10 (5.2%)	29 (13.9%)	
WHO-VAW – Psychological violence			
No	184 (96.3%)	7 (3.7%)	<0.001
Yes	138 (66%)	71 (34%)	
WHO-VAW – Physical violence			
No	190 (99.5%)	1 (0.5%)	<0.001
Yes	180 (86.1%)	29 (13.9%)	
WHO-VAW – Sexual violence			
No	190 (99.5%)	1 (0.5%)	0.009
Yes	199 (95.2%)	10 (4.8%)	
GHQ			
0–3	144 (62.9%)	85 (37.1%)	<0.001
≥4	47 (27.5%)	124 (72.5%)	

WHO-VAW, World Health Organization Violence Against Women; GHQ, General Health Questionnaire.

**Table 3:** Associations of psychological violence in the past 12 months with demographic, socioeconomic, obstetric and lifestyle characteristics, physical and sexual violence in the past 12 months, any lifetime violence and mental health changes of the pregnant women

Variable	No	Yes	*P*-value
Marital status			
Married/common-law union	296 (91.9%)	54 (69.2%)	<0.001
Single/divorced/widow	26 (8.1%)	24 (30.8%)	
Use of illicit drugs			0.028
No	318 (98.8%)	4 (1.2%)	
Yes	74 (94.4%)	4 (5.1%)	
WHO-VAW – Physical violence			
No	320 (99.4%)	2 (0.6%)	<0.001
Yes	50 (64.1%)	28 (35.9%)	
WHO-VAW – Sexual violence			
No	321 (99.7%)	1 (0.3%)	<0.001
Yes	68 (87.2%)	10 (12.8%)	
AAS			
No	184 (96.3%)	7 (3.7%)	<0.001
Yes	138 (66%)	71 (34%)	
GHQ			
0-3	210 (91.7%)	19 (8.3%)	<0.001
≥4	112 (65.5%)	59 (34.5%)	

WHO-VAW, World Health Organization Violence Against Women; AAS, Assessment Abuse Screen; GHQ, General Health Questionnaire.

Table 4 shows two multivariable logistic regression models. Any lifetime violence was the dependent variable in the first model, and psychological violence in the past 12 months was the dependent variable in the second model. In model 1, any lifetime violence was positively associated with being single (*P* = 0.005). The risk of a single woman experiencing violence was 2.95 times higher than that of a married woman. Regarding parity, no pregnancy or three or more pregnancies were positively associated with any lifetime violence (*P* < 0.001). Pregnant women who had experienced any lifetime violence were more likely to have mental health changes (odds ratio 4.67) compared with pregnant women with no history of violence (*P* < 0.01). In model 2, psychological violence in the past 12 months was also associated with being single. Women with a history of psychological violence were 5.93 times more likely to have mental health changes than pregnant women with no reported violence in the past 12 months.

**Table 4:** Associations of any lifetime violence and psychological violence in the past 12 months with the factors investigated: multivariable logistic regression models

Variables	Odds ratio (95% CI)	*P*-value
Model 1: violence (any lifetime)		
Age (years)		
<20	0.15 (0.717–1.900)	0.534
>30	0.59 (0.195–1.575)	0.268
20–30	Reference	
Marital status		
Single/divorced/widow	2.95 (1.345–5.765)	<0.01
Married/common-law union	Reference	
Per capita income^a^		
<0.5	1.60 (0.182–0.770)	0.388
0.5–1	1.43 (0.867–2.361)	0.161
>1	Reference	
Parity		
0	3.19 (1.383–7.396)	<0.01
≥3	3.54 (1.454–8.660)	0.01
1–2	Reference	
GHQ		
≥4	4.67 (2.900–6.345)	<0.01
0–3	Reference	
Model 2: psychological violence (past 12 months)		
Age (years)		
<20	1.35 (0.721–2.537)	0.347
>30	0.52 (0.095–2.896)	0.459
20–30	Reference	
Marital status		
Single/divorced/widow	4.92 (2.809–8.897)	<0.001
Married/common-law union	Reference	
Per capita income^a^		
<0.5	1.12 (0.326–3.89)	0.850
0.5–1	0.77 (0.419–1.423)	0.407
>1	Reference	
Parity		
0	1.30 (0.537–3.148)	0.421
≥3	2.03 (0.752–5.492)	0.09
1–2	Reference	
GHQ		
≥4	5.93 (2.783–9.104)	<0.001
0–3	Reference	

GHQ, General Health Questionnaire.

a. Minimum Brazilian wage of 1 is equal to US$267.00.

## Discussion

In this study, more than half of the pregnant women experienced violence at any time in their lives, and psychological violence was the most prevalent type (19.5%). A similar prevalence of psychological violence (22.2%) was reported in a study conducted in south-western Ethiopia on 590 pregnant women.^22^ Another study carried out in Jordan during the COVID-19 pandemic, involving 215 pregnant women, showed a prevalence of psychological violence of 50.2% among all types of violence.^23^ In a systematic review with meta-analysis of studies conducted during the COVID-19 pandemic, the combined prevalence of violence was 22% (95% CI 4–40%), with psychological, physical and sexual violence accounting for 24, 14 and 6%, respectively. The authors concluded that the prevalence of violence against pregnant women was higher during the pandemic than before this period.^24^ Psychological violence was also the most prevalent type (92.9%) in a study carried out on 830 Iranian pregnant women;^25^ along with sexual violence (11.0%), these types of violence showed the largest increase compared with the periods before pregnancy and before the COVID-19 pandemic.

To our knowledge, this is the first Brazilian study that investigated the prevalence of violence against pregnant women and its associated factors during the pandemic. The results of multivariable logistic regression analysis showed that any lifetime violence and psychological violence in the past 12 months were associated with being single. The findings are consistent with the meta-analysis conducted by James et al^4^ involving 140 287 women, in which being single during pregnancy had an odds ratio of 1.73 for domestic violence. A study of 379 Brazilian pregnant women carried out before the COVID-19 pandemic showed that unstable unions, single women and women as household head were risk factors for violence.^5^

Both types of violence investigated in this study – any lifetime violence and psychological violence in the past 12 months – were associated with mental health changes as measured by the GHQ. Mental health changes have been associated with a higher risk of exposure to domestic violence.^26^ Pregnant women who experience domestic violence tend to start antenatal care later and be at higher risk of depression, anxiety, and suicide ideation and attempt.^4^ The COVID-19 pandemic has demonstrated an effect on mental health. Pregnant women screened during the COVID-19 pandemic exhibited more distress (symptoms of anxiety and depression) and other psychiatric symptoms than those screened before the pandemic.^27^ A recent meta-analysis showed that the COVID-19 pandemic increased the risk of anxiety among women during pregnancy and during the perinatal period.^28^ In China, domestic violence raised the risk of prenatal anxiety and depression among pregnant women, and women who experienced mental violence were 3.55 times more likely to have a risk of depression.^29^

This study showed that maternal age was neither associated with any lifetime violence nor with psychological violence in the past 12 months. The finding was surprising, given that other studies conducted before the pandemic indicated that younger women are more vulnerable to psychological violence in the past 12 months.^5,30^ This difference may be explained by the fact that the prevalence of young women was only 7.5% in our study, compared with 23.5% and 16.8% in the studies by Audi et al^5^ and Vasconcelos et al,^30^ respectively.

Pandemic-induced economic recessions were found to be a predictor of violence against 590 pregnant women in a cross-sectional study carried out during the pandemic in south-western Ethiopia.^22^ On the other hand, a study with 830 Iranian pregnant women showed that high socioeconomic status was a risk factor for general violence.^25^ According to a review by Hunnicut,^3^ gender equality is measured based on socioeconomic indicators. If gender equality increases, the woman can either experience less violence as her status improves or more violence as a result of being victimised by the aggressor. In our study, socioeconomic status of the pregnant women was not associated with violence. This finding can be explained by the fact that almost all women were of low socioeconomic status, with the population being highly homogenous.

A strength of our study was that we screened for mental health and violence throughout the period of the pandemic. Another highlight was the training of the interviewers, who throughout the process were attentive to safety and created strategies when the participants were unable to answer the questions, including suggesting another time for the interview, ensuring that the aggressor was not with the participant and applying safety procedures according to individual reality. However, the cross-sectional design was a limitation of the study. Furthermore, because of the COVID-19 pandemic, we were unable to follow up on the pregnant women included in the study.

Moreover, it is important to note that violence during pregnancy may be underreported,^29^ since the data are obtained by self-report. Many pregnant women may avoid talking about their experience with violence. Guilt may be one of the reasons responsible for the inability to talk or even think about the event, which tends to manifest as mental and physical suffering. According to the WHO, ethics and safety in violence research is important for the participants and interviewers involved.^31^ In some cases, women only disclose violence when they feel safe and trust the interviewer.^32^ Moreover, the questionnaires depend on the memory of pregnant women, who may have forgotten or ignored violent events; this is especially true for psychological violence, which is characterised by invisibility, trivialisation and naturalisation in contemporary culture.^3^

In conclusion, several measures can be used to prevent domestic violence, especially training health professionals involved in prenatal care in the early detection of single women and women with mental health changes, who presented with a three to six times higher risk of experiencing violence. In addition, the study also detected other risk factors for domestic violence, such as no parity or having three or more parities, with risks 3–3.5 times higher for these women. It is also important to emphasise that more than half of the pregnant women had experienced violence at some point in their lives, and most of them had mental health changes. There is, therefore, an urgent need to address the issue of domestic violence during antenatal care and probably after childbirth, considering that domestic violence may also affect the health of offspring.

## Data Availability

Electronic copies of survey data are available from the corresponding author, P.H.C.R., on reasonable request.

## About the authors

**Leonardo Domingos Biagio** is a Master's student at the School of Public Health, University of São Paulo, Brazil. **Delanjathan Devakumar** is Professor of Global Child Health at University College London, UK. **Leticia Falcão de Carvalho** is a PhD student at the School of Public Health, University of São Paulo, Brazil. **Natália Pinheiro de Castro** is a post-doctoral researcher at the School of Public Health, University of São Paulo, Brazil. **Rossana Verónica Mendoza López** is a researcher at the Centre for Translational Oncology Research at the São Paulo State Cancer Institute (ICESP), Brazil. **Liania Alves Luzia** is a researcher at the School of Public Health, University of São Paulo, Brazil. **Perla Pizzi Argentato** is a post-doctoral researcher at the School of Public Health, University of São Paulo, Brazil. **Patrícia Helen Carvalho Rondó** is a professor at the School of Public Health, University of São Paulo, Brazil.
